# Molecular assays for the detection of prostate tumor derived nucleic acids in peripheral blood

**DOI:** 10.1186/1476-4598-9-174

**Published:** 2010-07-02

**Authors:** Matthias Jost, John R Day, Ryan Slaughter, Theodore D Koreckij, Deanna Gonzales, Martin Kinnunen, Jack Groskopf, Harry G Rittenhouse, Robert L Vessella, Mark A Reynolds

**Affiliations:** 1 Gen-Probe Incorporated, San Diego, CA 92121, USA; 2 Department of Urology, University of Washington, Seattle, WA 98195, USA; 3 Puget Sound VA Health Care System, Washington, DC 20420, USA

## Abstract

**Background:**

Prostate cancer is the second leading cause of cancer mortality in American men. Although serum PSA testing is widely used for early detection, more specific prognostic tests are needed to guide treatment decisions. Recently, the enumeration of circulating prostate epithelial cells has been shown to correlate with disease recurrence and metastasis following definitive treatment. The purpose of our study was to investigate an immunomagnetic fractionation procedure to enrich circulating prostate tumor cells (CTCs) from peripheral blood specimens, and to apply amplified molecular assays for the detection of prostate-specific markers (PSA, PCA3 and TMPRSS2:ERG gene fusion mRNAs).

**Results:**

As few as five prostate cancer cells were detected per 5 mL of whole blood in model system experiments using anti-EpCAM magnetic particles alone or in combination with anti-PSMA magnetic particles. In our experiments, anti-EpCAM magnetic particles alone exhibited equivalent or better analytical performance with patient samples compared to a combination of anti-EpCAM + anti-PSMA magnetic particles. Up to 39% of men with advanced prostate cancer tested positive with one or more of the molecular assays tested, whereas control samples from men with benign prostate hyperplasia gave consistently negative results as expected. Interestingly, for the vast majority of men who tested positive for PSA mRNA following CTC enrichment, their matched plasma samples also tested positive, although CTC enrichment gave higher overall mRNA copy numbers.

**Conclusion:**

CTCs were successfully enriched and detected in men with advanced prostate cancer using an immunomagnetic enrichment procedure coupled with amplified molecular assays for PSA, PCA3, and TMPRSS2:ERG gene fusion mRNAs. Our results indicate that men who test positive following CTC enrichment also exhibit higher detectable levels of non-cellular, circulating prostate-specific mRNAs.

## Introduction

Serum PSA testing is widely used for prostate cancer screening, however more specific tests are needed to guide treatment decisions following definitive biopsy. Furthermore, tests are needed to detect disease recurrence following radiation and/or surgical intervention, especially considering the increasing rate of targeted therapies for patients who do not have their prostates surgically removed. Considerable effort has been directed toward the development of methods for detecting circulating prostate tumor cells (CTCs) as an early indicator of distal disease progression. Early studies focused on RT-PCR methods for detection of prostate-specific mRNAs in whole blood [1,2]. These mRNAs were originally presumed to be a surrogate measure of the presence of CTCs, however conflicting results have been reported regarding the clinical utility of this approach [3,4]. More recently, investigators have focused on methods for detecting and enumerating CTCs directly [5], and one commercial assay is now available [6]. Nonetheless, the full clinical significance of CTCs remains somewhat controversial, although increasing numbers of clinical studies have supported this approach [7].

Circulating tumor cells have been isolated and characterized from the blood of cancer patients by a variety of methods [8]. The enumeration of CTCs in a population of advanced stage prostate cancer patients has been correlated with poor prognosis [9-11]. Furthermore, enumeration of CTCs following surgical intervention showed a greater correlation with survival than serum PSA monitoring [7].

Here we describe an immunomagnetic method of CTC enrichment that can be used as a convenient preanalytical step for detecting prostate-specific mRNAs by using transcription-mediated amplified (TMA) molecular assays. The method employed a standardized, magnetic particle-based capture system that is compatible with the automated TMA assay formats. Magnetic particles were derivatized with antibodies targeting either prostate specific membrane antigen (PSMA) or the epithelial cell adhesion molecule (EpCAM). PSMA, a type II membrane-bound glycoprotein, is mainly expressed in prostate tissue, although it has also been found in neovasculature [12,13]. Because its expression is elevated in prostate cancer tissues [14], we chose it to be an antigen for the immunomagnetic enrichment procedure described herein. We tested magnetic particles derivatized either with anti-EpCAM alone or with a combination of anti-EpCAM plus anti-PSMA to investigate potential synergy in CTC enrichment experiments.

Prostate-cancer-specific molecular markers have been reviewed recently and it is evident that the field will continue to evolve as new recurrent molecular markers are elucidated [15-17]. For the present work, we chose PCA3 mRNA and TMPRSS2:ERG gene fusion mRNA, since diagnostic utility has already been demonstrated for these markers in urine specimens [18,19]. We hypothesized that the immunomagnetic enrichment method described herein could be used together with PCA3 and TMPRSS2:ERG assays to specifically detect CTCs in advanced prostate cancer. TMPRSS2:ERG gene fusions have been associated with aggressive prostate cancer in a transgenic mouse model [20], detected in distant metastasis [21] and also linked with aggressive prostate cancer phenotypes in humans [22,23]. Moreover, it was demonstrated recently that a portion of TMPRSS2:ERG positive tumors did not respond to androgen ablation therapy [24]. We also included an amplified molecular assay for PSA mRNA with CTC enrichment as a marker for prostate-derived cells. The current study describes preliminary clinical data in support of feasibility for detecting the above molecular markers in CTCs and in most cases also in matched plasma specimens. Future studies will be needed to assess the clinical utility of the described assay system.

## Materials and methods

### Cell lines

The C4-2 prostate cancer cell line [25] was a gift from Leland Chung, Ph.D. (Emory University). LNCaP cells [26] were obtained from the American Type Culture Collection (ATCC; Manassas, VA). GFP-LNCaP cells were kindly provided by Dr. Srivastava, Center for Prostate Disease Research (Rockville, MD). All cell lines were grown under standard culture conditions in RPMI 1640 medium supplemented with 10% fetal bovine serum (ATCC), and with G418 (0.5 mg/mL; Invitrogen, Carlsbad, CA) for GFP-LNCaP cells.

### Preparation of prostate cancer cells for use in model system of immunomagnetic cell isolation

GFP-LNCaP cells were treated by mild trypsinization (Trypsin/EDTA, Invitrogen, Carlsbad, CA) from one 25 mm^2 ^dish (BD, Franklin Lakes, NJ) and harvested by sedimentation (500 g, 5 min, room temperature). The cell pellet was resuspended in PBS (dPBS, Invitrogen, Carlsbad, CA). C4-2 cells were harvested as described above for GFP-LNCaP cells. Individual cells (n = 5, 10, 25) were aspirated with a micromanipulator pipette system (TransferMan NK, Eppendorf and Leica DMIRB inverted microscope) and added to normal donor blood treated with EDTA to prevent coagulation. Freshly harvested LNCaP cells were transferred to a 10 cm Petri dish containing RPMI 1640 medium at room-temperature. Individual cells were aspirated manually with a standard laboratory pipette under low microscopic magnification (Olympus CK2 equipped with 10× lens) and added to EDTA-treated normal donor blood. Immunomagnetic cell isolation was carried out as described in more detail below. Briefly, for each of the cell lines tested individually in the model system, freshly resuspended cultured cells were added to 1 mL EDTA blood (approximately 100 cells) and incubated with 10 uL anti-EpCAM coated magnetic particles (Invitrogen, Carlsbad, CA) for 20 min at room temperature. Magnetic-bound fractions were washed three times, as described below, and then the washed particles were subjected to fluorescence microscopy as described below.

### Specimen processing

Specimen processing included CTC enrichment and cell lysis. For specimens that contained C4-2 cells, specimen processing was performed at the University of Washington (Seattle, WA) and the processed specimens were shipped to Gen-Probe Incorporated (San Diego, CA) on dry-ice for further testing. Processing of specimens that contained LNCaP cells or GFP-LNCaP cells was done at Gen-Probe Incorporated.

### Microscopy

For visualization experiments of magnetically enriched cells, about 20 μL Mowiol mounting medium [27] was added to the washed magnetic particles. The resulting suspension was spotted onto a glass slide and covered with a round glass cover slip (18 mm diameter) to prevent drying. The mounted cells were visualized with a fluorescence microscope with 10× and 40× lenses (Axio Imager Z1, Zeiss, Thornwood, NY). Pictures were taken with an attached CCD camera (Metasystems, Waltham, MA). GFP-LNCaP cells in EDTA-treated blood were visualized after spreading a thin film onto a glass slide without a cover slip to prevent cellular rupture.

### Patient Selection

Specimen collection was carried out at the University of Washington under IRB approved protocols that included written informed consent from each of the subjects. This study included consecutive patients who consented and fell within the inclusion and exclusion guidelines. In total, sixty-five men diagnosed with prostate cancer participated in this study: thirty-five men who had been diagnosed with advanced-stage prostate cancer by bone scan (hereafter referred to as advanced PCa) and twenty-nine who had been diagnosed as early-stage cancer patients by biopsy following a serum PSA determination and digital rectal exam (hereafter termed pre-radical retropubic prostatectomy (pre-RP)). Moreover, five men diagnosed with benign prostatic hyperplasia (BPH) were included in the study. Five age-matched healthy individuals served as controls for specificity of cell capture and molecular analysis. Blood from apparently healthy volunteers was used as a matrix for experiments in the model system (described above) that tested samples containing known amounts of cultured C4-2, LNCaP, and GFP-LNCaP cells.

### Collection of blood specimens

Whole blood was collected by venipuncture into two 10 ml EDTA-containing Vacutainer^® ^tubes (BD, Franklin Lakes, NJ) and stored on ice up to 4 hours before processing. The collected blood was pooled and then processed by immunocapture and plasma generation as described in detail below. In cases when only one vacutainer tube could be obtained, the processing procedure was: (1) perform immunomagnetic cell capture on 5 mL blood with anti-EpCAM and anti-PSMA coated particles, (2) plasma preparation from up to 4 mL blood, and (3) perform immunomagnetic CTC isolation with anti-EpCAM coated particles by using the remaining whole blood if sufficient volume remained.

### Immunomagnetic cell isolation of CTCs from whole blood specimens

Anti-EpCAM coated magnetic particles were purchased from Invitrogen (Carlsbad, CA). Anti-PSMA coated particles were generated by reacting a monoclonal anti-PSMA antibody (Beckman Coulter) with human anti-mouse magnetic particles according to manufacturer's specifications (Invitrogen, Carlsbad, CA).

For CTC isolation, 5 mL or 7.5 mL of whole blood was incubated with 100 uL antibody-coated magnetic particles (anti-PSMA and/or anti-EpCAM) for 30 min at room temperature. Magnetic-bound fractions were subjected to three washing steps with phosphate buffered saline (dPBS; Invitrogen) containing 0.2% (w/v) BSA (Jackson Immuno Research, West Grove, PA) and subsequently treated with Gen-Probe lysis buffer. The immunomagnetically-enriched fractions were stored at - 20°C until mRNA isolation was performed (see below).

### Plasma processing

Blood specimens (up to 4 mL of EDTA-treated blood) were subjected to sedimentation at 1,600 g for 10 min at 4°C to generate plasma. The plasma fraction was carefully separated from the cellular fraction by standard laboratory pipetting with a 1 mL tip and mixed with an equal volume of Gen-Probe lysis buffer. The treated plasma was stored at -20°C until mRNA isolation was carried out (see below).

### Molecular Testing using TMA amplified assays

TMPRSS2 (T2):ERG gene fusion, PCA3 and PSA mRNAs were either detected qualitatively or quantitatively using assays that included the steps of magnetic target capture, transcription-mediated amplification (TMA), and a hybridization protection assay. Specifically, target mRNA was purified from immunomagnetically enriched and processed plasma fractions by hybridization to magnetic particles via target-specific oligonucleotides (target capture step), amplified by TMA, and detected by using target-specific acridinium ester (AE)-labeled probes (hybridization protection assay step) as described in [18]. Three T2:ERG splice variants were detected qualitatively, T2:ERGa, b and c [28], also known as Types III, I and VI, respectively [29]. Amplification primers for T2:ERG gene fusion mRNA were located in T2 exon 1 and ERG exon 4 (T2:ERGa), T2 exon 1 and ERG exon 2 (T2:ERGb), and T2 exons 1 and 2 and ERG exon 4 (T2:ERGc). The AE-labeled probes for each T2:ERG splice variant spanned the junction between T2 and ERG. Primers for PCA3 mRNA targeted exons 3 and 4, with the AE-labeled probe spanning the exon 3/4 junction. Primers for PSA mRNA targeted exons 2 and 3, with the AE-labeled probe spanning the exon 2/3 junction. PCA3 and PSA mRNAs were detected quantitatively (signal-to-noise set to 2-fold or greater), whereas T2:ERG was a qualitative assay (cutoff = 100,000 relative light units, RLUs). Calibrators and controls consisted of T2:ERG, PCA3 or PSA *in vitro *transcripts (IVTs) in detergent solution. The T2:ERGa, b and c IVTs were prepared from plasmids provided by A. Chinnaiyan (University of Michigan) [28]. IVT copy levels were calculated based on spectroscopic concentration determination using A_260 _measurements. Assays were performed at Gen-Probe Incorporated using its DTS^® ^400 Systems and assay protocol for reagent addition volumes and incubation times and temperatures as previously described in [18].

## Results

### Model system studies for the detection of prostate cancer cells

We developed an immunomagnetic particle capture system for the isolation of rare cells out of blood based on antibody coated magnetic particles directed to epithelial and prostate cell surface epitopes. To assess successful isolation of cultured prostate cancer cells from a mixture of normal blood cells, we used microscopic or molecular methods.

**Figure 1:**
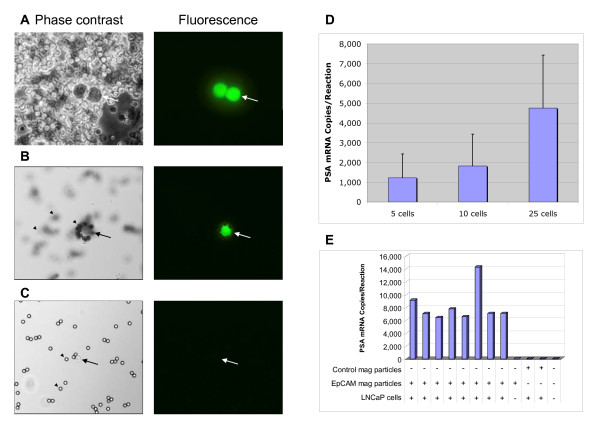
**CTC enrichment in model system experiments**. A) GFP-LNCaP cells mixed with blood from a normal donor were visualized by phase contrast and fluorescence microscopy using a 40× objective. Two fluorescent cells (see arrow) are visible in a background of non-fluorescent blood cells. B) GFP-LNCaP cell (see arrow) after enrichment with anti-EpCAM coated magnetic particles. Captured GFP-LNCaP cells were coated with numerous magnetic particles (non-fluorescent spheres, see arrowheads.) C) Immunomagnetic fraction from similarly processed normal donor blood (without added prostate cancer cells). This fraction is practically devoid of blood cells, with a low amount of non-fluorescent background material (see arrow). Magnetic particles are marked by arrowheads. D) PSA mRNA detection in immunomagnetically enriched C4-2 cells. Normal blood mixed individually in samples that contained 5, 10 and 25 added C4-2 cells were incubated and captured with anti-EpCAM magnetic particles out of 5 mL EDTA-treated blood. Error bars represent one standard deviation of repeat capture experiments (n = 10 for samples that contained 5 or 10 cells), n = 5 for samples that contained 25 cells). E) PSA mRNA copy numbers from immunomagnetically processed blood samples. Bars 1 through 8 represent replicate samples containing LNCaP cells (2 cells/mL, 4 mL total blood volume) that were processed using anti-EpCAM magnetic particles. Bars 9-12 represent control samples. The presence of LNCaP cells, control magnetic particles (devoid of anti-EpCAM primary antibody), or anti-EpCAM magnetic particles is indicated by the +/- symbols.

GFP-expressing LNCaP cells were added to normal donor blood and subsequently visualized before and after immunomagnetic enrichment (Figure 1A, and Figure 1B, C, respectively). As expected, the magnetic particle-bound fraction contained fluorescent GFP-LNCaP cells (Figure 1B) and only a small amount of non-fluorescent structures, possibly representing non-specifically bound blood cells (Figure 1C).

We next demonstrated molecular detection of prostate cancer cells with Gen-Probe's protocol that includes target capture, TMA and hybridization protection assay steps. PSA mRNA was detected in five C4-2 cells that had been immunomagnetically enriched from 5 mL of normal donor blood. The measured PSA mRNA copy numbers correlated well with cellular input (Figure 1D).

To investigate the specificity of immunomagnetic capture, we mixed LNCaP cells with normal donor blood and subjected the mixture to the enrichment steps using control magnetic particles that were devoid of anti-EpCAM or anti-PSMA primary antibodies. No PSA mRNA was detected in this condition, indicating that cell capture was antibody dependent (Figure 1E). Moreover, PSA mRNA was undetectable in magnetically enriched fractions from normal blood donors that were devoid of added cultured prostate cancer cells (Figure 1E). Both anti-EpCAM and anti-PSMA coated magnetic particles gave equivalent performance in these model system experiments (data not shown).

### PSA mRNA detection in CTC enriched fractions and plasma from prostate cancer patients

We next investigated the CTC enrichment method with freshly drawn blood specimens from prostate cancer patients. Samples were processed on site within four hours from time of collection. For those specimens with sufficient blood volume, we processed the specimens in three fractions: (1) CTC enrichment using anti-EpCAM plus anti-PSMA magnetic particles on whole blood; (2) CTC enrichment using anti-EpCAM magnetic particles alone on whole blood; and (3) plasma fraction using standard centrifugation. For specimens with insufficient blood volume, we omitted the second fraction (anti-EpCAM magnetic particles alone). Processed fractions were then tested using amplified molecular assays.

**Figure 2:**
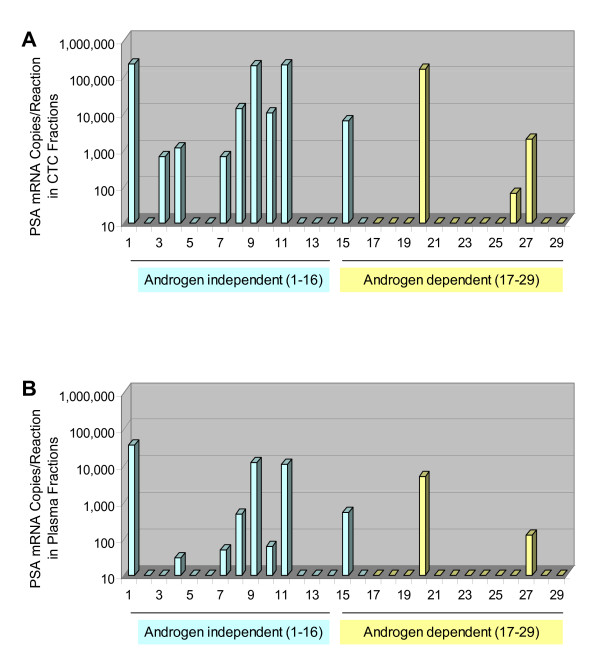
**PSA mRNA copy numbers in fractionated blood samples from prostate cancer patients**. A) CTC enriched blood fractions processed using the dual antibody (anti-PSMA plus anti-EpCAM) magnetic particle formulation. B) Matched plasma fractions.

Results for the PSA mRNA assay are summarized in Figure 2. As shown in Figure 2A, the combined anti-EpCAM plus anti-PSMA magnetic particle formulation detected 9/16 androgen-independent (56%) and 3/17 androgen-dependent samples (18%). BPH and early stage prostate cancer specimens were devoid of detectable PSA mRNA (0/5 and 0/29, respectively). Results from matched plasma fractions are shown in Figure 2B. In this case, we detected 8/16 androgen-independent (50%) and 2/17 androgen-dependent specimens (12%). Positive plasma fractions were in almost complete concordance with CTC enriched fractions, although CTC enrichment generally gave higher PSA mRNA copy numbers.

**Figure 3:**
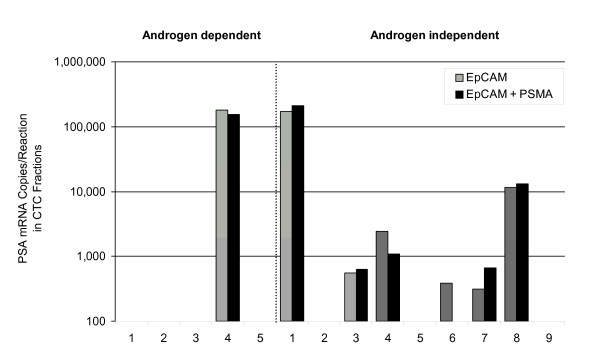
**Comparison of matched CTC enriched blood fractions using either dual antibody (anti-PSMA plus anti-EpCAM) or single antibody (anti-EpCAM alone) magnetic particle formulations**.

Figure 3 shows a comparison of PSA mRNA signals from CTC enriched samples where sufficient blood volume had been collected from the patient to allow a comparison between the single antibody (anti-EpCAM) and dual antibody (anti-EpCAM plus anti-PSMA) magnetic particle formulations. The single antibody formulation detected 8/14 cases (57%); whereas the dual antibody formulation detected 7/14 cases (50%), with the majority of positives from androgen-independent patients. We were unable to measure any synergistic effect of the dual antibody magnetic bead formulation in this experiment (see Discussion).

### Comparison of three different molecular markers in CTC enriched fractions and plasma from prostate cancer patients

A comparison of PSA, PCA3 and TMPRSS2:ERG mRNA signals is shown in Tables 1 and 2 for blood samples from men with advanced prostate cancer that were processed using the dual antibody magnetic particle formulation. Results from men with androgen-dependent prostate cancer are summarized in Table 1. These men all tested negative for PCA3, whereas one of the men was positive for TMPRSS2:ERG gene fusion mRNA. In contrast, more of the men with androgen-independent prostate cancer tested positive for all three markers (Table 2). PCA3 was positive in 5/16 (31%) of these patients, although PCA3 mRNA copy numbers were at least one order of magnitude lower than PSA mRNA copy numbers in these patients. Three of these patients tested positive for TMPRSS2:ERG gene fusion mRNA in CTC enriched samples (Table 2) and also in plasma (data not shown). When any of the three markers were positive the detection rate increased to 63% (10/16 positive; Table 2). This demonstrates feasibility of applying a research prototype TMA-based molecular assay to CTC enriched patient samples.

**Table 1:** Comparison of PSA, PCA3 and TMPRSS2:ERG mRNA copy levels in CTC enriched fractions from androgen-dependent prostate cancer patients.

Patient #	PSA	PCA3	TMPRSS2: ERG
1	-	-	N/A

2	-	-	N/A

3	-	-	N/A

4	154,893	-	-

5	-	-	-

6	-	-	-

7	-	-	-

8	-	-	-

9	-	-	-

10	66	-	-

11	1,928	-	+

12	-	-	-

13	-	-	-

14	-	-	-

15	-	-	-

16	-	-	-

17	-	-	-

Blood samples from prostate cancer patients were processed using the dual antibody (anti-PSMA plus anti-EpCAM) magnetic particle formulation. CTC enriched fractions were prepared from patients diagnosed with advanced androgen-dependent prostate cancer (n = 17). N/A = insufficient sample available for measurement.

**Table 2:** Comparison of PSA, PCA3 and TMPRSS2:ERG mRNA copy levels in CTC enriched fractions from androgen-independent prostate cancer patients.

Patient #	PSA	PCA3	TMPRSS2: ERG
1	213,277	9,851	N/A

2	-	-	N/A

3	631	-	-

4	1,097	-	-

5	-	312	-

6	-	-	-

7	658	-	-

8	13,294	-	+

9	189,634	546	-

10	10,088	4,478	-

11	208,727	-	+

12	-	-	-

13	-	-	-

14	-	-	-

15	6,264	261	+

16	-	-	-

Blood samples from prostate cancer patients were processed using the dual antibody (anti-PSMA plus anti-EpCAM) magnetic particle formulation. CTC enriched fractions were prepared from patients diagnosed with advanced androgen-independent prostate cancer (n = 16). N/A = insufficient sample available for measurement.

## Discussion

The present study was designed to assess the feasibility of an immunomagnetic enrichment method for detecting circulating prostate tumor cells using research prototype TMA assays for prostate-specific mRNAs (PSA, PCA3 and the TMPRSS2:ERG gene fusion). Detection of PCA3 and/or TMPRSS2:ERG mRNA in whole blood could potentially be used as a prognostic indicator of aggressive prostate cancer. Enumeration of prostate CTCs alone provides minimal information of the metastatic nature of these cells, although the presence of CTCs in blood would not be expected in the case of benign disease. Indeed, an increasing number of studies have demonstrated a correlation between prostate CTC numbers and survival following surgical intervention [7,9,30].

Microfluidic cartridges have been described for enriching CTCs from whole blood with high efficiency [31]. These cartridges contained anti-EpCAM antibodies coupled to spatially defined pillars in the device that were shown to efficiently capture CTCs in model system experiments. Interestingly, the authors of this study also reported enumeration of CTCs in early stage prostate cancer patients, with positive detection in about 90% of cases investigated. Other studies also reported detection of CTCs in early stage prostate cancer using anti-EpCAM magnetic beads combined with PCR [32]. In contrast, CTC enumeration using the Veridex CellSearch™ System failed to detect any CTCs in the blood of early stage prostate cancer patients [33], whereas this system detected up to 65% of patients with progressive, metastatic, castration-resistant disease [34].

PCA3 mRNA expression is elevated over 60-fold in prostate cancer tissues compared to benign tissues [35]. PCA3 to PSA mRNA expression ratios are increased in urine specimens from men with positive biopsy [18,36], therefore it seemed reasonable to assume that similar expression ratio differences could be used for molecular subtyping of prostate CTCs in blood specimens. Unfortunately, the positivity of the research prototype PCA3 assay used in these experiments was relatively low (less than 30% of androgen-independent prostate cancer specimens), and there were insufficient numbers of patient samples for a clinical correlation.

TMPRSS2:ERG gene fusion mRNAs have been associated with aggressive prostate cancer morphology in tissues [37] and a preliminary study using a combination of TMPRSS2:ERG plus PCA3 assays has shown synergistic diagnostic utility in urine specimens [19]. A preliminary investigation of TMPRSS2:ERG mRNA transcript levels in blood specimens of prostate cancer patients has been reported [38]. Although the authors reported detection of TMPRSS2:ERG gene fusions in 6/10 of the CTC enriched samples by FISH analysis, TMPRSS2:ERG mRNA transcripts could not be detected by RT-PCR. In contrast, the TMA-based assay gave relatively high signals for TMPRSS2:ERG mRNA in CTC enriched fractions from a subset of men with advanced androgen-independent prostate cancer. It should be noted that the prevalence of TMPRSS2:ERG gene fusions in prostate cancer is 44-50% in serum PSA-screened cohorts and 15-36% in population-based cohorts [39,40]. In metastatic disease the prevalence of TMPRSS2:ERG gene fusions was found to be similar to that observed in organ-confined prostate cancer [21,41]. In the present study, the prevalence of TMPRSS2:ERG gene fusions was about 21% (3 out of 14 androgen-independent donors, see Table 2), which is within the range of published studies [39,40].

As stated above, percentages of positive CTC detection vary among published studies in advanced prostate cancer patients. It should be possible to improve detection rates for PCA3 and TMPRSS2:ERG in CTC enriched fractions by increasing the analytical sensitivity of these assays. For example, the PCA3 assay used in the present study was developed originally for urine specimens [18]. A more sensitive version of the assay is currently under development for use with blood specimens. Positive detection rates could also be improved by using larger sample volumes. Regardless, the detection rates reported here are encouraging and suggest that molecular stratification of advanced prostate cancer patients is feasible. Additional studies are needed to validate the utility of this approach.

PSMA is known to be over-expressed in advanced prostate cancer or castration resistant prostate cancer [14]. We were unable to measure a significant difference when comparing anti-EpCAM magnetic beads versus our dual-antibody magnetic beads. This was true for both androgen-dependent and androgen-independent sub-populations (Figure 3). In one case, a patient was positive with the anti-EpCAM magnetic beads but not with the dual-antibody magnetic beads. This could simply be a stochastic difference when splitting a sample containing very dilute numbers of CTCs. Clearly, larger numbers of patient specimens would need to be tested for a significant comparison.

The detection of prostate-specific mRNAs in patient plasma is not a new finding, however it is interesting to note that our results for plasma testing showed a high correlation with results obtained when CTC enriched fractions were tested, suggesting that patients who shed high numbers of CTCs into blood have similarly high levels of prostate-specific circulating mRNAs. Not surprisingly, the CTC fraction exhibited higher copy numbers as compared to the corresponding plasma fraction. In the case of CTCs, 5-7.5 mL of whole blood was enriched and tested, whereas a smaller volume of plasma was tested without enrichment. Plasma samples are more accessible and easier to work with than CTC enrichment from fresh blood. This finding was also reported by Helo et al. using other genetic markers [42], where plasma levels of KLK3, KLK2, and PSCA mRNA showed a high correlation with CTC numbers using the CellSearch™ assay. To our knowledge, the present study is among the first to report a similar correlation using amplified molecular assays for both plasma and CTC enriched fractions, and suggests that molecular subtyping of CTCs is feasible in advanced prostate cancer patients.

The nature of circulating prostate-specific mRNAs is worthy of further study. Recent studies suggest that they could be encapsulated in circulating exosomes that are shed from invasive prostate tumors [43]. It has been hypothesized that this is one mechanism by which prostate cancer cells can sensitize the immune system [44]. Further study of exosome fractions in prostate cancer patients is warranted to determine whether this is a clinically informative sample fraction.

In summary, a method for immunomagnetic enrichment of prostate CTCs from patient whole blood specimens has been described. This method is compatible with automation, and is particularly compatible with amplified assays based on TMA for detection of PSA, PCA3, and TMPRSS2:ERG mRNAs. It also provides an objective result without the use of cytometry or imaging. The automated sample processing method is beneficial for testing the large numbers of patient specimens that would be needed for further clinical association studies.

## Competing interests

The authors declare that they have no competing interests.

## Authors' contributions

MJ participated in the design and coordination of the study, developed and tested the cell capture method with prostate cancer cell lines, conducted data analysis, and drafted the manuscript. MAR directed the design and coordination of the study, contributed to methods development and assay designs, and contributed to drafting the manuscript. RLV directed the patient accrual and assisted with the design of the study. HGR and JG assisted with study design. RS adapted prototype molecular assays for testing plasma samples and conducted molecular testing at Gen-Probe Incorporated. JRD directed the adaptation of the prototype molecular assays, coordinated the molecular testing of the study samples, and conducted data analysis. TK, DG and MK performed experiments at the University of Washington. All authors read and approved the final manuscript.
